# The Addition of Endometrial Injury to Freeze-All Strategy in Women with Repeated Implantation Failures

**DOI:** 10.3390/jcm10102162

**Published:** 2021-05-17

**Authors:** Ioannis Rigos, Vasileios Athanasiou, Nikolaos Vlahos, Nikolaos Papantoniou, Dimitrios Profer, Charalampos Siristatidis

**Affiliations:** 1 Assisted Reproduction Unit, IVF Athens Center, Leof. Kifisias 5, 15123 Athens, Greece; athanassiou@ivfathenscenter.gr; 2 Assisted Reproduction Unit, Second Department of Obstetrics and Gynecology, “Aretaieion Hospital”, Medical School, National and Kapodistrian University of Athens, Vas. Sofias 76, 11528 Athens, Greece; nfvlahos@gmail.com (N.V.); csyristat@med.uoa.gr (C.S.); 3 Third Department of Obstetrics and Gynecology, “Attikon Hospital”, Medical School, National and Kapodistrian University of Athens, Rimini 1, Chaidari, 12642 Athens, Greece; npapant@gmail.com; 4 Independent Researcher, Pontou 9-11, 62125 Serres, Greece; dimitrisprofer@gmail.com

**Keywords:** endometrial injury, hysteroscopy, freeze-all strategy, repeated implantation failures, in vitro fertilization (IVF), pregnancy rates

## Abstract

(1) Background: Recurrent implantation failure (RIF) after IVF remains a challenging topic for fertility specialists and a frustrating reality for patients with infertility. Various approaches have been investigated and applied towards the improvement of clinical outcomes. Through a nonrandomized clinical trial, we evaluated the effect of the combination of hysteroscopic endometrial injury and the freeze-all technique on pregnancy parameters in a cohort of RIF patients; (2) Methods: The study group comprised of 30 patients with RIF that underwent a hysteroscopic endometrial injury prior to a frozen embryo transfer cycle; another 30 patients with RIF, comprising the control group, underwent a standard frozen cycle with no adjuvant treatment before. Live birth comprised the primary outcome. Logistic and Poisson regression analyses were implemented to reveal potential independent predictors for all outcomes. (3) Results: Live birth rates were similar between groups (8/30 vs. 3/30, p = 0.0876). Biochemical and clinical pregnancy and miscarriages were also independent of the procedure (*p* = 0.7812, *p* = 0.3436 and *p* = 0.1213, respectively). The only confounding factor that contributed to biochemical pregnancy was the number of retrieved oocytes (0.1618 ± 0.0819, *p* = 0.0481); (4) Conclusions: The addition of endometrial injury to the freeze-all strategy in infertile women with RIF does not significantly improve pregnancy rates, including live birth. A properly conducted RCT with adequate sample size could give a robust answer.

## 1. Introduction

Infertility remains a major issue affecting human reproduction globally [1]. In developed countries, up to 16.7% of the population suffers from the disease, and more than half of the couples seek advice for assisted reproduction treatments (ART) [2]. Although the efficacy of these has been improved, clinical pregnancy rates per embryo transfer after IVF do not exceed a 33.2% threshold [3]. The main step of treatment success is embryo implantation, which depends on endometrial receptivity, the quality of the embryo, and individual exogenous factors, including endometriosis, hydrosalpinx, and suboptimal ovarian stimulation [4]. In many cases, despite the availability of top-quality embryos, implantation does not occur [5]. Various parameters have been associated with implantation failure, and interventions usually aim at addressing the underlying disease.

Recurrent implantation failure (RIF) indicates the absence of implantation after repeated good-quality embryo transfers. Although this clinical problem is frequently encountered and there is vast associated literature, there is no universally accepted definition. Most published articles define RIF as the failure to achieve a clinical pregnancy after a minimum of two to six previous fresh or frozen cycles [6]; others define RIF as the failure to achieve a clinical pregnancy after a minimum of three IVF cycles with at least four good-quality embryos in a minimum of three cycles in women under 40 years old [7]. The heterogeneity of the definitions reflects inconsistencies in the number of failed embryo transfers and the number and quality of embryo(s) transferred, as well as the definition of this pathological entity, per se.

Two of the most commonly proposed, used, and studied interventions to overcome RIF are endometrial injury (EI) and the “freeze-all” strategy. Both of them aim to improve unexplained defective endometrial receptivity. The positive effect of the former was initially reported 15 years ago [8,9] in nonrandomized trials. The pathophysiologic mechanisms include an increase in the secretion of cytokines, interleukins, and growth factors; the differentiation of immune cells into macrophages or dendritic cells; and the restoration of uterine natural killer cells [10,11,12,13]. The results from clinical trials are conflicting [9,14,15,16,17,18,19,20,21,22,23,24,25,26,27,28,29,30,31,32,33,34]. As for the latter, the intervention aims to restore the asynchrony between the endometrium and the embryo status, which has been suggested to be caused by high steroids’ levels due to ovarian stimulation [35]; thus, the endometrial maturation stage appears to be more “advanced” at the time of the embryo transfer [36,37]. In this concept, and through the evolution of vitrification, elective freezing of all embryos followed by subsequent frozen–thawed transfer cycles were reported as effective in improving ART results, providing a more “physiological” endometrial environment [35,38,39,40,41].

In the absence of current robust data in the literature, we scheduled a combined approach in RIF patients. Thus, through a nonrandomized controlled trial, we aimed to investigate whether the addition of EI before a frozen–thawed cycle of a “freeze-all” strategy can improve pregnancy outcomes in this particular category of infertile patients.

## 2. Materials and Methods

This is a two-center two-arm nonrandomized clinical study conducted at the Assisted Reproductive Units of the Third Department of Obstetrics and Gynecology, “Attikon” Hospital, National and Kapodistrian University of Athens, Athens, Greece, and the IVF Athens Center, a private fertility clinic in Athens, Greece; patients were recruited from March 2017 to July 2018 and February 2018 to December 2019 in the two units, respectively. All patients were previously diagnosed with infertility and RIF. All patients met the criteria for undergoing a controlled ovarian hyperstimulation (COH) protocol followed by IVF/ICSI, after a detailed medical history and clinical evaluation. The trial protocol was approved by the Scientific Board and Bioethics Committee of the “Attikon” Hospital [Approval Number: 4/13–3-17 (2140/21–2-17)] and the Scientific Board of IVF Athens Center [Approval Number: 201801/1 (08/01/18)].

### 2.1. Patient Population/Eligibly Criteria and Study Design

The initial study was registered at https://clinicaltrials.gov/ (ClinicalTrials.gov ID: NCT04597463; accessed on 2 April 2021). Although the original study design was to be a prospective randomized study (RCT), most patients, before their enrollment in the randomization process, preferred to receive the intervention. As a result, randomization was not feasible, and relevant data were preferably analyzed as a nonrandomized clinical study in order to assess the preset outcomes of the study, although in an altered nature.

No preimplantation genetic screening or diagnosis was performed for any of the patients enrolled. The following demographic and laboratory parameters were also recorded: age, BMI, smoking status, hormonal profile (basal FSH, E2, LH, AMH, PRL, and TSH), parity, AFC, type (primary/secondary) and duration of infertility, semen analysis, IVF cycle, and embryo characteristics. All previous IVF/ICSI cycles were performed in the two IVF Units. A written informed consent form was obtained from all of the participants following consultation in plain language and before the initiation of the intervention protocol.

The inclusion criteria for the study entry were as follows: history of failure to achieve a clinical pregnancy after a minimum of 3 failed embryo transfers with high quality embryos; age ≤ 42 years; BMI ≥ 20 and ≤30; basal FSH ≤ 15 mIU/mL; absence of any known and/or untreated immunological or hematological disorders (through thrombophilia and antiphospholipid antibodies screening and parental karyotype); normal endometrial cavity at hysteroscopy or HSG. Exclusion criteria included: age > 42 years; BMI < 20 and >30; basal FSH > 15 mIU/mL; presence of severe endometriosis (stage III–IV); and medical history of adnexectomy or oophorectomy.

For the fresh cycle, both the GnRH antagonist and the long-21 GnRH agonist protocols were used. The study group comprised of 30 patients with RIF who underwent an hysteroscopic EI preceding the embryo transfer cycle. EI was performed in the early proliferative phase of the cycle with three cuttings of 0.5 cm on the front endometrial wall, 1 cm lower than the endometrial fundus level, while no antibiotics were administered. A 5.0 mm continuous flow rigid scope with 30-degree view and normal saline as distention media were used. The control group was comprised of patients with RIF and underwent a standard frozen cycle with no adjuvant treatment before the embryo transfer. Both EI and embryo transfers were performed by the same skilled and trained gynecologists (CS and VA), using a nontouch approach and transabdominal ultrasonography, respectively. The allocation of the participants in the two Units followed a principle of 1:2, according to the number of cases that each one treated per year. Participants were then enrolled in the study following a 1:1 principle.

### 2.2. Frozen Replacement Protocol

For the hormone replacement cycle, patients received exogenous estradiol valerate (Cyclacur, Bayer Hellas A.B.E.E.), 3 tabs orally starting from day 2 of the cycle until the endometrial thickness was >7 mm, where transvaginal administration of micronized progesterone was added, either in the form of vaginal suppositories (Utrogestan, Basins Iscovesco, Paris, France, vaginal tablets, 200 mg t.i.d), or vaginal gel (Vasclor, Vag.Gel 8%, Verisfield UK, Ltd., Chalandri, Greece, twice daily).

### 2.3. Selection of Embryos and Embryo Transfer

Selection of embryos for transfer was performed through an identical protocol in both units, in order to ensure a homogeneous result; it was based on their developmental rate and morphological features, both assessed by light microscopy. Embryo quality was assessed according to morphological criteria based on the overall blastomere number, size, appearance, and degree of fragmentation [42]. For each patient, embryos of the best quality were selected for transfer. These were conducted at day 3 or 5 (following a 1:1 principle and decided according to the quality of embryos at day 3), while the maximum number of embryos transferred was two, as in accordance with the Hellenic legislation.

### 2.4. Outcome Measures

The primary outcome measure was live birth, while secondary outcome endpoints were biochemical, clinical pregnancy and miscarriage rates, and number of sacs. Definitions of the outcomes were according to the International Glossary on Infertility and Fertility Care [43].

### 2.5. Statistical Analysis

Our primary analysis was performed to provide a direct comparison between groups. All endpoints, besides the number of sacs, were binary (yes/no) and expressed by their frequencies. Number of sacs, describing counts, is described by its median and range. Initial mean and proportion comparisons were made using a t-test, z-test and Mann–Whitney test. The independence of the categorical endpoints with the EI was assessed with the chi-square test. Fisher’s exact test was performed wherever the expected frequency of the cases was <5. All tests were two sided. The independence of the number of sacs with the EI was examined with a Poisson regression model, since the variables in question were counts and categorical.

In order to reveal potential independent predictors that contributed to the change of the primary outcomes, we planned to perform logistic and Poisson regression analysis. Logistic and Poisson regression models, depending on the endpoint, were further employed in order to examine possible confounding effects from three additional variables, (i) BMI, (ii) female age, and (iii) number of retrieved oocytes. Lastly, a sensitivity analysis was performed only on the primary endpoint. The same logistic Regression model was used with the inclusion of the categorical variable day of embryo transfer (3 or 5). Statistical significance was set at a significant level of 0.05. All statistical analyses were performed in SAS software, version 9.4.

## 3. Results

### 3.1. Study Characteristics

The initial number of participants that met the inclusion criteria and were recruited for the study was 72, but due to withdrawals and failures to follow up, sufficient data for analysis was obtained for 60 of them: half of them constituted the study and the rest the control group, respectively.

Table 1 contains descriptive statistics for the main patients’ demographic and baseline characteristics. A Shapiro–Wilk test was implemented to check their normality. For those found to be normal, mean and standard deviation was reported, and a t-test was used to make mean comparisons between groups. For the non-normal ones, median and range were reported instead, and the comparisons were made using a Mann–Whitney test. All differences were found to be nonsignificant.

### 3.2. Primary Analysis of the Primary and Secondary Outcomes

Proportion comparisons for the endpoints are shown in Table 2. All comparisons were made by using a z-test, except for the number of sacs where a Mann–Whitney test was employed. All comparisons returned nonsignificant results.

### 3.3. Secondary Analyses

Table 3 contains the results of the chi-square test of independence. In the cases of biochemical pregnancy and miscarriage, the combinations between the categories of the variables resulted in very small expected frequencies in the cells of the contingency tables. Therefore, the chi-square test would not be appropriate, and as such, the Fisher’s exact test was also employed in order to compare the results.

The two tests agree in their respective results and no significance was observed. All endpoints have no association with EI before embryo transfer, which means that whether a patient had her endometrium injured or not had no effect on whether her pregnancy resulted in a live birth or not. Similar conclusions can be extracted for the rest of the binary outcomes (biochemical pregnancy, clinical pregnancy, miscarriage).

Since the nature of the number of sacs FHB was counts, it could not be directly tested in terms of its association with the EI before embryo transfer, which is categorical. For this reason, a Poisson regression model was employed, with the number of sacs as the dependent variable and the EI before embryo transfer as the independent variable. Based on the results in Table 4, there is no association present.

During the logistic and Poisson regression analyses, every endpoint was modeled with the EI before embryo transfer and each one of the confounding factors. Only one situation returned a significant result, the endpoint of biochemical pregnancy, and the significant confounder was the number of retrieved oocytes (*p*-value = 0.0481, 95% CI: (0.0014, 0.3223)). For every additional oocyte that is retrieved, the odds of having a positive result on pregnancy hCG increase by e0.1618 ≈ 1.17 times. The rest of the results were found to be nonsignificant (Table 5).

There was no significant difference in the distribution between the two groups, according to the day of transfer (z-test, Table 6). Sensitivity analysis returned slightly altered results, but the direction of the effect estimate remained the same (Table 7).

## 4. Discussion

The purpose of this two-center two-arm nonrandomized clinical study was to compare the effect of the addition of endometrial injury (EI) to freeze-all cycles in infertile patients with RIF, in terms of pregnancy rates. The study group underwent a hysteroscopic EI preceding the embryo transfer cycle, while the control group received no additional intervention. The outcomes examined included laboratory and clinical parameters following a frozen replacement cycle, including maternal side effects. We found similar live birth rates between the two groups. Our results demonstrated that whether a patient had her endometrium injured or not, this had no beneficiary effect on live birth rate, while similar conclusions were extracted for the rest of the secondary endpoints, namely biochemical and clinical pregnancy, miscarriage, and number of sacs, as none of them manage to reach statistical significance. The only confounding factor that contributed to biochemical pregnancy was the number of retrieved oocytes.

Surprisingly, there is insufficient evidence so far in the literature concerning the effect of EI in frozen only cycles. In our study, patients with laboratory or clinical findings of endocrinologic disorders or endometrial pathology were excluded in order to be certain that any observed beneficial effect would be associated with the intervention. The hypothesis behind the selective inclusion of women with RIF was that a combined benefit of EI and the “freeze-all” policy would aid this category of patients.

For the use of EI on patients undergoing IVF/ICSI, the Cochrane systematic review of Nastri [44], encompassing 14 trials and 2128 patients, concluded that EI performed between day 7 of the previous cycle and day 7 of the embryo transfer cycle seems to improve live birth and clinical pregnancy rates in women with RIF, but evidence is, as stated by the authors, “of moderate-quality”, so more well-designed trials are necessary in order to stratify the results for women with RIF and report live birth. Interestingly, the use of office hysteroscopy only, with or without corrected abnormalities, may be of positive prognostic value for improving pregnancy outcomes in patients with RIF [45]. Possible explanations for this have been analyzed in a recent systematic review [46]. In this review, the authors, including 10 studies, found significant higher live birth and clinical pregnancy rates when EI was applied, while the difference disappeared when they included only studies with a low risk of bias, in which the age of participants was over 30 years, and in which no hysteroscopy was performed for the intervention [46].

For the use of the “freeze-all” policy, a systematic review and meta-analysis [35], including three trials with 633 IVF cycles in women aged 27–33 years, concluded that frozen embryo transfers resulted in significantly higher ongoing and clinical pregnancy rates. The Cochrane systematic review comparing a “freeze-all” strategy with conventional IVF/ICSI [47] showed that one strategy is not superior to the other in terms of cumulative live birth rates, though, as the authors stated, evidence was of moderate to low quality due to serious risk of bias and imprecision. Currently, there is a lack of scientific evidence to support the widespread use of the strategy for indications such as implantation failure, high progesterone levels on the trigger day, endometriosis, or advanced maternal age.

Data on the combination of EI and the “freeze-all” strategy are very limited. In our study, the hysteroscopic EI before a frozen cycle in patients with RIF did not manage to exhibit a positive effect on both the primary and secondary outcomes set for this study. We did not expect such results, as the combination of the two methods has been reported to improve clinical pregnancy rates [24,48,49]. Even in these studies, differences from ours are apparent. Matsumoto et al., in a quasi RCT, included 77 RIF patients, using an Endocyte^®^ sampler during the luteal phase of the cycle preceding the one that was used for blastocyst transfer; the authors found increased clinical and reduced miscarriage rates [24]. Similarly, Kanazawa et al., in a retrospective cohort study, reported increased clinical and ongoing pregnancy rates in a cohort of 173 RIF patients when double mild curettage was applied in the menstrual cycle prior to blastocyst transfer, irrespective of their quality, but not in a group that underwent hysteroscopy only [48]. Finally, similar results were described in a large case-control study with patients with one previous implantation failure, by inserting a biopsy catheter during the proliferative phase (day 5 of menstruation) of the FET cycle [49]. Thus, the different ways of performing EI render direct comparison with our results rather difficult.

In the presence of these studies, we made the assumption that the addition of EI to a freeze-all strategy would improve live birth, replicating the reported positive effects for patients with RIF. The differences observed did not manage to reach statistical significance. Likewise, two prospective studies ended up with no differences in clinical pregnancy rates, where a pipelle or an endometrial sampler device was used during the luteal phase before preceding to the embryo transfer cycle [30,50]: the authors acknowledged the possibility of a null effect, as hysteroscopy was not implemented, while they emphasized that the conflicts with other studies’ results are probably due to the differences in design, methods, type of samples encompassed, and sample size.

### Limitations and Strengths

The results of this study do not aim to provide a clinical policy. The strength of the study lies in its prospective design, with a priori set of parameters for the evaluation of two methods used in patients with RIF and the use of live birth as the primary outcome. The predetermined dataset was extensive and detailed, representing all clinical steps during the application of EI before the thawed embryo transfer. The limitations of the study are mainly attributed to its nature. The lack of randomization and blinding are linked with possible unknown confounders and selection bias. Although we planned to conduct an RCT, that was not feasible, due to practical reasons. Most participants having already experienced the disappointment of RIF denied being randomly enrolled in the study, hoping that the intervention might be beneficial. Another limitation is the reduced cohort size, justified by the observed tendency surrounding the application of the intervention, without the risk of allocation in the control group. Thus, we might observe statistically higher live birth rates favoring the intervention, instead of the marginal significance (*p*-0.0876) we found in our study with the specific cohort size. Moreover, due to the small cohort size, there were unequal distributions of the day 5 embryos (60% vs. 53%); although the z-test for the 60% vs. 53% difference returned a p-value of 0.6022, the difference decreased the robustness of the results. We did not proceed to the calculation of the sample size; instead, our aim was to follow the number of participants of previous studies [50,51]. We acknowledge the small sample size used in our cohort; however, a sample size of 60 is adequate to detect a difference of 30% in live birth or clinical pregnancy rates (post hoc analysis), which is acceptable for IVF studies using EI as a treatment method. In fact, in the study by Dunne and Taylor [50], the authors detected a difference in clinical pregnancy of 37%, while in a previous study from our group [51], we found a difference of 20% in live birth rate, which is similar to the results from the current study (we detected a difference of 16.7% in live birth rate). Moreover, the study was prematurely ended because of the COVID-19 pandemic: IVF protocols in all units worldwide changed from fresh to frozen and patients were denied the participation in a study where an intervention was offered, so it was impossible to recruit more patients during the last two years. Finally, as explicitly stated, no preimplantation genetic screening or diagnosis was performed for any of the patients enrolled: that could have led to the possible transfer of aneuploid embryos in this cohort of patients. Thus, the lack of power calculation renders the final result less robust and should be considered with caution; a proper sample size calculation based on the primary outcome of this study (live birth) would give more accurate answers to the specific question. Additionally, in order to explore more deeply the effect of the specific intervention and to offer more robust conclusions, a control group of “endometrial injury and fresh cycle” might be of relevance, along with preimplantation genetic testing of all embryos transferred.

## 5. Conclusions

The addition of EI to a frozen cycle in a “freeze all” strategy in infertile women with RIF does not significantly improve pregnancy rates, including live birth. A well-designed RCT should be performed with adequately described randomization and allocation concealment methods to provide a robust answer to this particular clinical question.

## Figures and Tables

**Table 1:** Mean and median differences of the main study characteristics.

**Normal Variables**	**Study Group**	**Control Group**	**Mean** **Difference**	***p*-Value**
Age at Cycle	34.90 (4.32)	34.97 (3.83)	0.07	0.9498
E2	63.63 (23.77)	68.99 (35.40)	5.37	0.4938
MII Oocytes	6.93 (2.93)	5.63 (2.55)	1.30	0.0723
2PN	5.60 (2.27)	4.63 (2.01)	0.97	0.0858
Sperm Motility	0.40 (0.17)	0.41 (0.15)	0.01	0.7320
**Non-Normal Variables**	**Study Group**	**Control Group**	**Median Difference**	***p*** **-Value**
BMI Level	22.15 (10.70)	22.66 (13.54)	0.51	0.6734
No of Retrieved Oocytes	8.00 (15.00)	7.00 (14.00)	1.00	0.2059
No of Previous Miscarriages	0.00 (2.00)	0.00 (3.00)	0.00	0.3770
No of Previous IVF Attempts	2.00 (4.00)	1.00 (4.00)	1.00	0.5116
FSH	7.20 (10.10)	7.21 (7.60)	0.01	0.4419
LH	6.08 (19.10)	5.37 (7.15)	0.71	0.1187
AMH	24.67 (61.28)	22.48 (56.08)	2.19	0.3632
PRL	15.96 (29.52)	20.30 (28.60)	4.34	0.1373
TSH	2.31 (3.78)	2.48 (3.85)	0.17	0.8766
Stimulation Days	11.50 (7.00)	12.00 (4.00)	0.50	0.2508
Total Gonadotrophins	2550.00 (2725.00)	2337.50 (3000.00)	212.50	0.5584
AFC	10.00 (15.00)	9.00 (15.00)	1.00	0.4096
Age at Menarche	13.00 (8.00)	12.00 (6.00)	1.00	0.0759
Sperm Concentration	33,500,000 (97,000,000)	30,000,000 (148,000,000)	3,500,000	0.4410
Sperm Morphology	0.97 (0.07)	0.98 (0.07)	0.01	0.2689
Sperm Concentration After Activation	10,000,000 (49,500,000)	6,500,000 (19,500,000)	3,500,000	0.3537
Sperm Motility After Activation	0.80 (0.65)	0.90 (0.63)	0.10	0.1555

**Table 2:** Proportion comparisons for all endpoints.

Variable	Study Group	Control Group	Difference	*p*-Value
Live Birth	8/30 (26.67%)	3/30 (10.00%)	16.67%	0.0876
Biochemical Pregnancy	10/30 (33.34%)	9/30 (30.00%)	3.34%	0.7812
Clinical Pregnancy	8/30 (26.67%)	5/30 (16.67)	10.00%	0.3436
Miscarriage	2/30 (6.67%)	6/30 (20.00%)	13.33%	0.1213
Number of Sacs	0.00 (2.00)	0.00 (1.00)	0.00	0.2839

**Table 3:** Chi-square and Fisher’s tests. *: Expected frequencies < 5.

Variables	Chi-Square Value (*p*-Value)	Fisher’s Test (*p*-Value)
Live Birth	2.7829 (0.0953)	0.1806
Biochemical Pregnancy	0.0770 (0.7814)	0.9999
Clinical Pregnancy	0.8838 (0.3472)	0.5321
Miscarriage *	2.3077 (0.1287)	0.2542

**Table 4:** Poisson regression model for number of sacs FHB in the group that undewent endometrial injury [(Endom. Inj. (Y)] before ET.

Variables	Parameter Estimate	Standard Error	Wald χ^2^ Value	*p*-Value
Endom. Inj. (Y)	0.7885	0.5587	1.99	0.1582

**Table 5:** Logistic and Poisson regression results (*p*-Values with an asterisk indicate statistical significance). The bold numbers are used to indicate statistical significance.

Variables	Parameter Estimate	Std. Error	Wald χ^2^ Value	*p*-Value
**LIVE BIRTH**				
Endom. Inj. (Y)	1.1845	0.7363	2.59	0.1077
BMI Level	−0.0492	0.1168	0.18	0.6736
Endom. Inj. (Y)	1.1868	0.7356	2.60	0.1067
Age at Cycle	0.0132	0.0837	0.03	0.8742
Endom. Inj. (Y)	1.0680	0.7495	2.03	0.1541
No. of Retr. Ooc.	0.1315	0.0936	1.97	0.1602
**Biochemical PREGNANCY**				
Endom. Inj. (Y)	0.1498	0.5578	0.07	0.7883
BMI Level	−0.0647	0.0969	0.45	0.5042
Endom. Inj. (Y)	0.1522	0.5564	0.07	0.7844
Age at Cycle	−0.0271	0.0690	0.15	0.6940
Endom. Inj. (Y)	−0.0295	0.5846	0.01	0.9598
No. of Retr. Ooc.	**0.1618**	**0.0819**	**3.91**	0.0481 *
**CLINICAL PREGNANCY**				
Endom. Inj. (Y)	0.5956	0.6417	0.86	0.3534
BMI Level	−0.0491	0.1089	0.20	0.6521
Endom. Inj. (Y)	0.6009	0.6418	0.88	0.3491
Age at Cycle	0.0367	0.0791	0.22	0.6427
Endom. Inj. (Y)	0.4378	0.6646	0.43	0.5101
No. of Retr. Ooc.	0.1597	0.0892	3.20	0.0734
**MISCARRIAGE**				
Endom. Inj. (Y)	−1.2638	0.8647	2.14	0.1439
BMI Level	−0.0642	0.1400	0.21	0.6466
Endom. Inj. (Y)	−1.2772	0.8681	2.16	0.1412
Age at Cycle	−0.0737	0.0985	0.56	0.4542
Endom. Inj. (Y)	−1.4476	0.8959	2.61	0.1062
No. of Retr. Ooc.	0.1313	0.1084	1.47	0.2257
**No. OF SACS**				
Endom. Inj. (Y)	0.7785	0.5396	2.08	0.1491
BMI Level	−0.0798	0.0893	0.80	0.3714
Endom. Inj. (Y)	0.7862	0.5394	2.12	0.1450
Age at Cycle	−0.0212	0.0606	0.12	0.7268
Endom. Inj. (Y)	0.6338	0.5459	1.35	0.2456
No. of Retr. Ooc.	0.1259	0.0653	3.72	0.0537

**Table 6:** Distribution of day 3 and day 5 embryo transfers.

Day of Embryo Transfer	Study Group	Control Group	Difference	*p*-Value
Day 3	12/30 (40.00%)	14/30 (46.67%)	6.67%	0.6022
Day 5	18/30 (60.00%)	16/30 (53.33%)	6.67%	0.6022

**Table 7:** Sensitivity analysis results.

Variables	Parameter Estimate	Std. Error	Wald χ^2^ Value	*p*-Value
**Live Birth**				
Endom. Inj. (Y)	1.2232	0.7408	2.73	0.0987
Day of ET (5)	0.4537	0.7089	0.41	0.5222
Endom. Inj. (Y)	1.2140	0.7408	2.69	0.1012
Day of ET (5)	0.4264	0.7129	0.36	0.5497
BMI Level	−0.0409	0.1159	0.12	0.7240
Endom. Inj. (Y)	1.2352	0.7418	2.77	0.0959
Day of ET (5)	0.6717	0.8306	0.65	0.4187
Age at Cycle	0.0542	0.1025	0.28	0.5972
Endom. Inj. (Y)	1.0837	0.7559	2.06	0.1517
Day of ET (5)	0.1256	0.7629	0.03	0.8692
No. of Retr. Ooc.	0.1260	0.0994	1.61	0.2047

## Data Availability

The data presented in this study are available on request from the corresponding author. The data are not publicly available due to privacy reasons.
